# Transvaginal Drainage of Pelvic Abscesses and Collections Using Transabdominal Ultrasound Guidance

**DOI:** 10.1155/2015/283576

**Published:** 2015-05-31

**Authors:** Kevin C. Ching, Jules H. Sumkin

**Affiliations:** Department of Radiology, University of Pittsburgh, 200 Lothrop Street, Suite 3950, Presby South Tower, Pittsburgh, PA 15213, USA

## Abstract

*Objectives*. To evaluate clinical outcomes following transvaginal catheter placement using transabdominal ultrasound guidance for management of pelvic fluid collections. *Methods*. A retrospective review was performed for all patients who underwent transvaginal catheter drainage of pelvic fluid collections utilizing transabdominal ultrasound guidance between July 2008 and July 2013. 24 consecutive patients were identified and 24 catheters were placed. *Results*. The mean age of patients was 48.1 years (range = 27–76 y). 88% of collections were postoperative (*n* = 21), 8% were from pelvic inflammatory disease (*n* = 2), and 4% were idiopathic (*n* = 1). Of the 24 patients, 83% of patients (*n* = 20) had previously undergone a hysterectomy and 1 patient (4%) was pregnant at the time of drainage. The mean volume of initial drainage was 108 mL (range = 5 to 570). Catheters were left in place for an average of 4.3 days (range = 1–17 d). Microbial sampling was performed in all patients with 71% (*n* = 17) returning a positive culture. All collections were successfully managed percutaneously. There were no technical complications. *Conclusions*. Transvaginal catheter drainage of pelvic fluid collections using transabdominal ultrasound guidance is a safe and clinically effective procedure. Appropriate percutaneous management can avoid the need for surgery.

## 1. Introduction

Percutaneous catheter drainage of abdominal and pelvic fluid collections is a well-established technique used by interventional radiologists for more than 30 years [1–3]. Sampling and drainage of organized fluid collections greatly affect clinical management as these collections may represent seromas, hematomas, or abscesses. Since transabdominal catheter drainage was first described in the 1980s, image-guided catheters are now being placed via transgluteal, transrectal, transperineal, and transvaginal routes using ultrasound and CT guidance [3–5]. While additional techniques for percutaneous catheter placement have become available, draining fluid collections within the pelvis remains a challenging task due to critical intervening structures. The urinary bladder and overlying bowel loops often prevent transabdominal access to fluid collections within the true pelvis. Sciatic neuropathy has been reported in 10% of patients following transgluteal catheter drainage [6]. Transvaginal (TV) catheter drainage of pelvic fluid collections was first described by Nosher et al. utilizing transabdominal (TA) sonography. [7]. A study of 14 patients by VanSonnenberg et al. described both TA and TV ultrasound guidance for transvaginal drainage of pelvic collections. Other than a few case reports, the authors of this paper could not locate a clinical study in the literature assessing patient outcomes following TV catheter placement using TA ultrasound [5, 7]. From our experience, advantages of TV catheter placement using TA ultrasound include excellent visualization of collections within the close confines of the true pelvis, the ability to select the exact location of vaginal puncture, and the ability to perform TV drainage when an endovaginal ultrasound transducer and/or needle guide is not available. This study reports the clinical efficacy and safety of our 5-year experience with transvaginal catheters placed using transabdominal ultrasound guidance for management of pelvic fluid collections.

## 2. Methods

### 2.1. Patient Population

The study was approved by the institutional review board and requirement for informed consent was waived. A single-center retrospective review was performed of all consecutive patients who underwent transvaginal drainage using transabdominal ultrasound guidance between July 2008 and July 2013 at a tertiary care women's hospital. Patients who underwent percutaneous drainage using CT guidance or transvaginal ultrasound were excluded. Drainage of nonorganized free pelvic fluid was also excluded. Our preference for the management of pelvic collections is to perform CT or US guided transabdominal drainage when feasible. When contraindicated by intervening structures, a transvaginal route is chosen for catheter drainage. While the decision for TA versus TV US guidance is ultimately attending dependent, almost all TV drainage procedures at our institution are performed using transabdominal US guidance. Antimicrobial therapy is administered prior to drainage in patients with clinical signs and laboratory findings of infection. The microbial culture results obtained from drainage result in discontinuation of antibiotics or tailoring of antibiotics for specific organisms. Patient demographics, procedure reports, clinical notes, and imaging studies were reviewed via the electronic medical record and picture archiving and communication system.

### 2.2. Study Outcomes

Clinical success was the primary outcome evaluated in this study. This was defined as clinical improvement without the need for surgery or additional percutaneous procedures to manage the pelvic fluid collection. Technical success and complications were studied as secondary outcomes as little data evaluating TA ultrasound for placement of TV catheters exists. Additional data was collected from the medical record including technical details, anesthesia, pelvic anatomy, and microbial specimen analysis.

### 2.3. Patient Demographics

A total of 24 patients underwent TV catheter placement using TA ultrasound guidance between July 2008 and July 2013. All pelvic fluid collections were diagnosed on computed tomography (CT) studies performed prior to the drainage procedures. The mean age of patients was 48.1 years (range = 27–76 y). In the 24 patients, 24 catheters were placed; however, 4 catheters were immediately removed after postprocedure US showed no residual fluid and the fluid was clear in appearance. All study participants except 1 were inpatients at the time of referral. Pelvic collections were postoperative in 21 patients (88%), were due to pelvic inflammatory disease in 2 patients (8%), and were idiopathic in 1 patient (4%). Of the 21 postoperative collections, 16 (76%) occurred following hysterectomy via transabdominal or transvaginal surgical approaches using traditional, laparoscopic, and robot-assisted techniques. The remaining 5 postoperative collections occurred following surgery for pelvic organ prolapse (*n* = 2), salpingectomy for ectopic pregnancy, laparoscopic appendectomy, and ventral hernia repair each (*n* = 1). Regarding pelvic anatomy, 20 of 24 patients (83%) no longer had a uterus and 1 patient was 11 weeks pregnant at the time of drainage.

### 2.4. Catheter Placement and Aspiration Procedures

**Figure 1:**
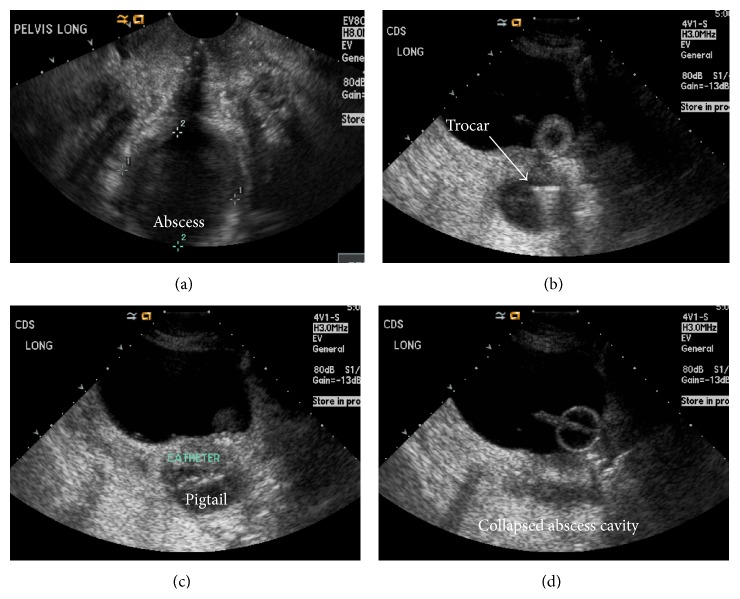
(a) Transvaginal ultrasound shows a small collection in the rectouterine space. (b) Sagittal transabdominal ultrasound shows the trocar puncturing inferiorly through the vaginal cuff. (c) 6 F pigtail catheter is advanced into the collection. 45 mL of pus was aspirated with collapse of the abscess cavity (d). The catheter was removed 2 days later and the patient was discharged. Cultures were positive.

For assessment of technical feasibility, all patients first underwent TA pelvic ultrasound. If the fluid collection was not well visualized, the urinary bladder was distended with approximately 250 cc of sterile saline to create a sonographic window and improve visualization. The patient was placed in the lithotomy position and a vaginal speculum was inserted. The vagina was then prepped with a povidone-iodine solution. Some patients received moderate conscious sedation when using intravenous midazolam and fentanyl was felt necessary. Using a 3–5 MHz linear ultrasound transducer, longitudinal TA sonography (Figure 1(a)) was performed to visualize the targeted pelvic fluid collection. Locking pigtail universal drainage catheters (Navarre, CR Bard, Inc.) ranging from 6 F to 10 F were placed using Trocar or Seldinger techniques. In our department an ultrasound technologist often scans the patient allowing the radiologist to use both hands for catheter placement; however, the procedure and sonography are easily performed by a single operator. The radiologist can identify the appropriate puncture position by indenting the vaginal mucosa with a blunt instrument such as ring forceps and identifying it with ultrasound. For the Trocar technique instillation of local anesthetic using a 27-gauge needle is made under real-time sonographic guidance, and then a 2 mm nick is made in the posterior fornix or vaginal apex with a standard length 11-blade scalpel. The assembled catheter system is inserted into the collection under real-time TA ultrasound guidance (Figure 1(b)). The inner trocar is removed allowing the pigtail to form and the catheter is then locked (Figure 1(c)). The Seldinger technique is performed similarly; however, initially a 7 inch 18-gauge Quincke spinal needle (BD, Franklin Lake, NJ) is first directed under TA ultrasound guidance into the collection and a 0.035 inch short Rosen J wire is placed through the needle into the collection. A vaginal nick with the scalpel is not necessary when using the Seldinger technique. While 23 of 24 procedures were performed using the Trocar technique, due to equipment availability in our department we use a cystography table so the wire exchange (Seldinger technique) can be performed with fluoroscopic assistance; however, this is not mandatory. The drainage catheter is then inserted over the wire and locked in place. Immediately following catheter placement, as much fluid is aspirated as possible through the catheter and postprocedure transabdominal sonography (Figure 1(d)) is performed to confirm catheter positioning and assess residual fluid. Since a locking catheter is placed, no suture or fixation device was used to secure the catheter. Technique for catheter placement, catheter size, and decision to leave a catheter in place were at the discretion of the treating radiologist. The volume of fluid removed was recorded in procedure notes and fluid specimens were sent for microbial culture. Timing of catheter removal was made by the clinical team and radiology physicians using dwindling catheter output (<10 mL in 24 hours) and clinical improvement as indications for discontinuation. Date of catheter removal was documented in the electronic medical record and the number of catheter days was recorded for the study.

## 3. Results

Diagnostic workup included CT imaging in all patients (100%) with additional pelvic ultrasound performed in 5 patients (21%). In 22 patients (92%), fluid collections were located in the mid deep (true) pelvis within the rectouterine space or rectovesical space. The remaining 2 collections (8%) were left adnexal.

Of the 24 catheters placed, 20 were left to gravity drainage and 4 catheters were immediately removed after sonography revealed sufficient drainage and the fluid was not infected in appearance. The 24 ultrasound guided procedures were performed by 5 attending radiologists. Drainage catheters placed were 6–10 F in size with 8 F being most common (*n* = 17, 85%). Bladder filling was performed through a Foley catheter in 58% of patients (*n* = 14) to create a sonographic window. This required Foley catheters to be placed in 11 patients that were immediately removed following the procedure. The remaining 3 patients had an indwelling bladder catheter already in place. 13 of 24 patients (54%) received additional moderate conscious sedation. The Trocar technique was employed for 23 of 24 (96%) catheter placements and the Seldinger technique was used for the remaining patient. Procedure notes documented the volume of fluid initially drained in 22 of 24 patients which averaged 108 mL (range, 5–570 mL). CT or ultrasound was performed in 2 patients (10%) 2 to 14 days following drainage to assess residual fluid prior to catheter removal. Seventeen specimens (71%) returned a positive culture. Catheters were left in place for an average of 4.3 days (range = 1–17 d).

Average length of follow-up was 572 days (range, 1-2,039 d) from the date of percutaneous drainage to the most recent gynecology note in the electronic medical record. All collections were successfully managed with single percutaneous drainage resulting in a clinical success rate of 100%. There were no technical complications. Inadvertent catheter dislodgement occurred in 3 of 20 patients (15%); however, none required catheter replacement or further intervention. No patients required surgery for management of pelvic fluid collections; however, 2 patients were taken to the operating room for repair of fascial dehiscence and debridement of an infected peritoneal chemotherapy catheter.

## 4. Discussion

Pelvic fluid collections are most commonly postoperative in etiology [1]. As gynecologic surgery is increasingly performed using minimally invasive techniques, open surgical drainage of infected collections would negate the benefits of laparoscopic surgery; therefore nonsurgical percutaneous management is the treatment of choice for draining pelvic collections [8]. Multiple prior studies have reported the efficacy of transvaginal catheter placement for management of pelvic fluid collections [1, 5, 7, 9]. Other than 5 reported cases, these studies utilize TV sonography for imaging guidance [5, 10]. In the study by VanSonnenberg et al. evaluating transvaginal catheter placement in 14 patients, TA ultrasound was used in the first two patients; however the authors noted better visualization with TV sonography which they used for the remaining 12 patients [5]. Distending the urinary bladder improves visualization within the pelvis by creating a sonographic window for TA ultrasound. This was not mentioned in that study. Refinements in ultrasound transducer technology in the 20 years since the reported cases also aid in visualization using the technique we describe.

While this study does not compare the results of TA versus TV ultrasound guidance, the authors believe that there are many benefits to our technique. Using the bladder as a sonographic window, we achieve excellent visualization of deep pelvic fluid collections even when they are small and closely adjacent to bladder and bowel. Also, because only the catheter is placed into the vagina, we are able to better choose our site of puncture through the vaginal wall. This may be helpful in posthysterectomy patients when the vaginal cuff is often friable due to an underlying infection. The pressure exerted by the endovaginal ultrasound probe on the sensitive and healing vaginal cuff when TV sonography was used is also avoided when TA ultrasound is utilized for catheter placement. Having utilized this procedure for many years, the authors believe it is important to be familiar with alternative techniques for transvaginal drainage in the event an endovaginal ultrasound transducer and/or needle guide is not available.

The main disadvantage of using transabdominal sonography for transvaginal drainage procedures is the need for a well-distended bladder. Foley catheters were placed in 46% of patients in our study for bladder filling. While no urinary tract infections (UTI) were documented, bladder catheterization is a risk factor for nosocomial UTI. Bladder distension may also be uncomfortable to patients. In a study comparing TV versus TA ultrasound for embryo transfer, bladder distension was associated with moderate to severe discomfort in 22% of patients [10]. While intraprocedural pain was not reported in our study, moderate IV sedation is well tolerated when additional analgesia is required. Lastly, while the equipment we describe is not available in all departments (cystography table), the majority of procedures in this study were performed using a Trocar technique which do not require fluoroscopy.

Limitations of our study include its retrospective design and lack of a comparison study group using a different method for image guidance. The small sample size also prevented a meaningful statistical analysis from being performed. Our department places transvaginal catheters using transabdominal and transvaginal imaging guidance. When transvaginal catheter placement is indicated the decision for TA versus TV ultrasound guidance is attending dependent. Of note, almost all TV catheters placed in our department are placed using TA ultrasound guidance. In our study the choice of catheter size was also variable ranging from 6 F to 10 F. This may have affected the number of catheter days and rate of clinical improvement as use of larger catheters has been associated with improved clinical outcomes when managing abdominal and pelvic fluid collections [11]. The rate of inadvertent catheter dislodgement was relatively high in our study at 15%; however there was no need for catheter replacement in any of the 3 patients. In addition, dislodgement of transvaginal catheters is an issue independent of whether TV or TA ultrasound guidance was used for the initial placement.

In conclusion, transabdominal sonography for placement of transvaginal catheters is a safe and effective variation of a proven technique for the management of pelvic fluid collections. Our clinical success rate of 100% is comparable to studies using TV ultrasound guidance as well as transgluteal and transabdominal routes for catheter drainage. Physicians may find this technique for TV catheter placement beneficial in postoperative hysterectomy patients or when an endovaginal transducer or needle guide is not available.
